# Cardiac Complications in Marfan Syndrome: A Review

**DOI:** 10.7759/cureus.29800

**Published:** 2022-09-30

**Authors:** Jayant Singh, Anil Wanjari

**Affiliations:** 1 Department of Medicine, Jawaharlal Nehru Medical College, Datta Meghe Institute of Medical Sciences, Wardha, IND

**Keywords:** tgf beta, cardiomyopathy, aortic dissection, cardiovascular complication, fibrillin 1

## Abstract

Marfan syndrome (MFS) is a rare inherited disorder of the connective tissue with an autosomal dominant mode of inheritance which happens as a result of a mutation in the fibrillin-1 (*FBN1*) gene located on chromosome 15q21.1. This mutation results in the defective formation of microfibrils and increased levels of active transforming growth factor beta (TGF beta), leading to defective connective tissue synthesis. These changes affect various parts of the body but most notably affected are the heart, eyes, and the musculoskeletal system. The standard presenting features of a person suffering from MFS are tall stature with a large arm span, kyphosis, congenital dislocation of the lens (ectopia lentis) and cardiovascular manifestations. The 2010 modified Ghent criteria are used to diagnose MFS on the basis of parameters such as cardiovascular, eye, and musculoskeletal disorders. The cardiovascular manifestations in a patient with MFS are the leading causes of mortality. The most common and dreaded complication is an aortic aneurysm and subsequent dissection. Cardiomyopathy and arrhythmia are also potential killers in such patients. This article aims to look at the various cardiac complications mentioned above and gain an understanding of their pathogenesis, incidence, and outcome. It also includes a brief overview of the rare complication post-Bentall graft infection, and its cause, diagnosis, and management. Various articles by several different authors from around the world were searched for information regarding the pathogenesis, incidence, and outcomes of these patients and are referenced below.

## Introduction and background

Introduction

Marfan syndrome (MFS) is one of the most common autosomal dominant disorders of the connective tissue, with a prevalence ranging from 1.5 to 17.2 per 100000 individuals [1]. There is no male or female sex predilection of the disease, thus seen with equal incidence in both males and females [2]. According to many pieces of research carried out independently across the globe by various researchers, it has been found that the most common etiological factor for the causation of the disease is a gene called fibrillin-1 (*FBN1*) gene [3]. It is situated on chromosome 15q21.1 and encodes a glycoprotein *FBN1*, the major component of the microfibrils situated in extracellular matrix [4]. As a result of the mutation, there are many manifestations in the eyes, cardiovascular system, and skeletal system, which are typical in a case of MFS. The clinical picture of the patient suffering from the disease is very typical, with the patient being very tall as a result of disproportionate growth of upper limbs and lower limbs, and the wingspan is more than 1.05 times the height of the person with elongated fingers [4]. The thoracolumbar vertebra also shows scoliosis, a sideward curvature of the spine in more than 60% of the patients diagnosed with this condition [5]. In ocular manifestations, this disease is characterised by ectopia lentis, which is congenital dislocation of the lens that is bilateral [6]. Cardiac manifestations also characterise the condition, the most characteristic of which is the dilation of the aortic root and proximal ascending aorta, which inevitably leads to aortic aneurysm and dissection in the early years of life that is under 40 years of age [7]. Late manifestations in the case of MFS may be aortic valve incompetence leading to aortic regurgitation [8]. The most common cause of death in patients with MFS is the result of aortic dissection with a case fatality rate of 1-3%, which happens in the early years of life [9]. The diagnostic criteria most widely accepted for MFS is called the revised Ghent nosology, which has major and minor criteria made after international expert opinion for accurately recognising the congenital condition to improve management and outcome of the patient [10].

Background

Genetic Basis

The main genetic defect associated with MFS is the *FBN1 *mutation found on chromosome 15q21.1. It is a large glycoprotein molecule with a molecular weight of 350 kDa, which is synthesised and released by fibroblasts and embedded into the extracellular matrix in the form of insoluble microfibrils [11,12]. These microfibrils serve as a foundation where elastin is deposited and are important for building a proper elastic framework for providing elasticity to dynamic connective tissues [13]. The structure of these fibrils is stabilised by the epidermal growth factor (EGF), which is arranged in tandem orientation [14]. These EGF prevent the fibrinolysis of the fibrils with the help of calcium-binding EGF (cbEGF). They act by sequestering the calcium in the extracellular compartment [15], thereby preventing their breakdown and promoting interactions between cellular components like αvβ3 and fibrillin monomers. Therefore, its action is by stabilizing the microfibril structure favouring lateral packing [16,17]. Thus, it can be said that mutations in fibrillin-1 result in a disordered as well as weak elastic fibre formation, which can lead to a microfibril network connection disruption between the adjacent cells [18]; it can be proposed that this mutation gives rise to abnormally weak wall of the vessels, which are prone to dissection and dilation, which can be due to the hemodynamic stresses and intraluminal pressure that is commonly observed in patients with M.F.S.

Apart from the theory mentioned above, another proposed mechanism related to elevated levels of transforming growth factor beta (TGF beta) is proposed in a paper by Dietze et al. [11]. According to the paper, inactive TGF beta molecules are first cleaved in the endoplasmic reticulum from where the active form is released [19]. The active form is sequestered with an inactive latency-associated peptide (LAP), which forms a latent biological product called a small latent complex (SLC). This SLC further covalently binds to latent TGF beta binding protein (LTBP) as a result of the formation of disulfide bonds between cysteine residues in the LAPs and LTBP [20]. This binding leads to the formation of a large latent complex (LLC) which is present in the extracellular space. This LLC then finally binds to the microfibrils produced by the *FBN1* gene. Now, since the microfibrils produced by the *FBN1* gene are defective due to a mutation in the gene, the binding of LLC to microfibrils is defective. This failure of binding leads to the failure of the inactivation of TGF beta in the extracellular compartment [21]. Another research article published by Chaudhary et al. showed the presence of a peptide on *FBN1* which is capable of disrupting the association of *FBN1* and C peptide of LTBP [22]. As a result of this dissociation between the two molecules, there is defective inactivation of TGF beta. This increases the levels of activated TGF beta in the extracellular compartment. All these factors together lead to increased levels of TGF beta, which can cause increased collagen deposition in the aortic wall. Increased collagen reduces the elastic property of the aortic wall and thus may lead to the wall becoming aneurysmal and dilated due to loss of structural integrity [21].

All the information in this article has been derived from numerous articles published on PubMed, which have been cited below in the references section. The search included keywords like "Genetic basis", "Cardiovascular manifestations" and "Treatment". Extracardiac manifestations were not included in the search or the article. The search included both review articles and clinical trials 

## Review

Complications

Aortic Aneurysm and Dissection

The most commonly encountered and the most characteristic feature of a patient suffering from MFS, which is also a major diagnostic criterion along with lens subluxation in the 2010 revised Ghent nosology, is the increase in the diameter of the aortic root. This dilation of the aortic root, which can ultimately lead to dreaded complications like aortic dissection and aneurysm, is the single most important and frequent reason of mortality in a patient with MFS [23]. The condition's prevalence is shown to be more in adults than in children and has been suggested in a study by Roman et al. according to which the prevalence of aortic root dilation is marginally higher in adults in comparison to children (approximately 90% vs 80%) [24]. The same was also shown by another study by Erkula et al., which showed the prevalence of aortic root dilation in adults and children to be 81% vs 76% [25]. In terms of sex predilection for the development of an aortic aneurysm, it is shown in many studies that males are generally more predisposed to the development of aortic aneurysm than females [24,26]. Similar results were observed for aortic dissection, which was more common in males than females. The cause for the aortic abnormalities can be attributed to the defective microfibrils addressed above as a result of *FBN1* mutation and TGF beta overactivity. These lead to defective aortic wall formation, which can cause aneurysm and dissection due to intraluminal pressure and hemodynamic stresses.

Another commonly observed abnormality seen in patients with MFS is valvular regurgitations, namely mitral valve regurgitation and aortic valve regurgitation. According to a study by Rybczynski et al., more than 50% of the patients with MFS report having regurgitation of the mitral valve. Out of those, severe regurgitation in the mitral valve was reported in up to 12% of the diagnosed patients by the age of 30 years [27]. Aortic valve regurgitation, which can be linked to the aortic valve annulus dilation as a result of *FBN1* gene mutation, is observed in up to one-third of adult patients [28]. This was shown in a study conducted by Porciani et al., in which mitral valve prolapse was seen in 78.9% of patients and mitral insufficiency in 72.7% of patients out of a total of 227 patients [28]. As a result of chronic valvular insufficiency, these patients are at an increased risk of developing chronic heart failure, which occurs as a result of an increase in end-diastolic volume and end-systolic volume, which may not be compensated adequately [1].

Heart Failure

Recent research has also shown the adverse effects of *FBN1* mutation on the contractility of the heart muscles. A study conducted by Yetman et al. among 70 patients with MFS with documented cardiac manifestations showed that 34 patients (68%) had a dilated left ventricle and eight patients (11%) had reduced ejection fraction [29]. This was also shown by De Backer et al. in their study, which showed that patients with MFS undergoing MRI revealed a reduction in the ejection fraction as well as in peak systolic velocities and an increase in the end-systolic volume [30].

Though many studies were done on the effect of MFS on left ventricles, not many studies were done regarding the effects on the efficiency of the right heart to pump blood. It changed when a study done by Kiotsekoglou et al. suggested, based on Doppler and conventional echocardiography, that in comparison to controls, MFS patients showed defective right ventricular systolic function, which was expressed as a reduced dp/dt ratio [31]. This was later confirmed by a study done by Alpendurada et al. It suggested that the ejection fraction of the right ventricle was reduced by 10.3%, with an increase in end-diastolic volumes of the right ventricle by 11.8%. Also, end-systolic volume was increased in 13.2% out of a total 68 patients with MFS [32]

Various tests were conducted on lab-bred mice regarding the role of *FBN1* gene mutation on MFS-related cardiomyopathy to gain a greater insight into the pathophysiology of MFS. The tests revealed that Fbn1 (C1039G/+) mice showed left ventricular contraction dysfunction. Subsequent observations of the heart muscle also revealed upregulation of the TGF beta-related molecular pathway, which was consistently associated with microfibril abnormalities in the mice [33]. Another study conducted by cook et al. suggested that Fbn1mgR/mgR mice synthesised around 20% of the usual amount of fibrillin-1. Such mice ultimately died from ruptured aortic aneurysms during the first year of life. Echo findings also showed significant aneurysms of the aortic root along with proximal ascending aorta and severe regurgitation in mitral and aortic valves [34]. This finding very much correlates to the findings seen in the patients suffering from MFS. This implicates the role of the *FBN1* gene in the pathogenesis of cardiac complications.

**Figure 1:**
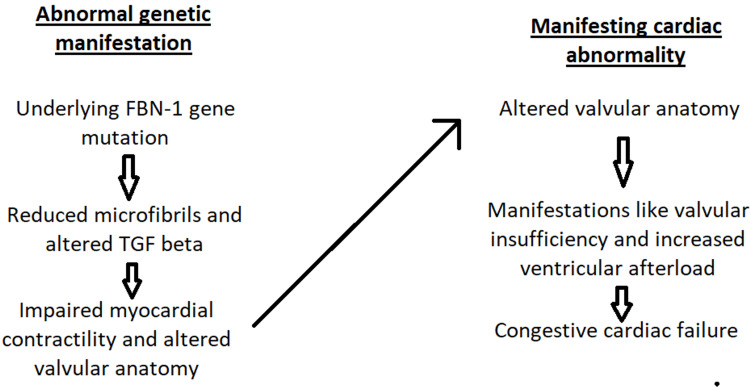
Flowchart showing genetic factor and its role in the pathophysiology of heart failure FBN-1 = fibrillin-1; TGF beta = transforming growth factor beta Image credit: Author Jayant Singh

Thus it can be said that microfibrils play a crucial role in the myocardium. They do so by maintaining the adequate compensated response of the myocardium to various stresses such as volume and pressure overload. In cases where microfibrils are defective, this compensation is lost, and thus, there can be manifestations leading to a reduction of ejection fraction by the heart [1]. The reduced ejection fraction of the heart can lead to an increase in the end-systolic volume and, subsequently, an increase in end-diastolic volume, which can precipitate heart failure, which is observed in patients with MFS The same is also shown in Figure 1.

Arrhythmia

Arrhythmia in the case of MFS is less likely to be seen in comparison to aortic root abnormality and cardiomyopathy, although it has been shown that people with *FBN1* gene mutation have a higher prevalence of ventricular arrhythmia by around 48% in comparison to normal people. This was attributed to mutations in exons 24-32 of the *FBN1* gene [35]. In a study by Muiño-Mosquera et al., out of total 86 patients suffering from MFS, 20 patients had non-sustained ventricular tachycardia (NSVT). Out of those, 12 patients were documented as having previous valvular surgery or valvular dysfunction [36]. It is also shown in many studies that patients with valvular abnormalities and insufficiencies are more at risk for developing arrhythmias [29]. Factor N-terminal pro-b-type natriuretic peptide (NTproBNP) has been implicated, independent of valvular pathologies [1]. It has also been implicated as an independent risk factor in a study by Hoffmann et al., which suggested that values more than 214.3 pg/ml of NTproBNP are associated with sustained ventricular tachycardia and sudden cardiac death [37]. Therefore it can be hypothesised that the presence of arrhythmia in a patient with MFS is mostly not a primary pathology. It is rather a secondary manifestation of cardiomyopathy or heart failure resulting from valvular abnormalities. Table 1 illustrates the results of three studies done by separate authors and their findings in regard to arrhythmia seen in patients with MFS.

**Table 1:** Table showing three studies and their results regarding the prevalence of arrhythmias in patients with Marfan syndrome (MFS) ECG = electrocardiography; 2D echo = two-dimensional echocardiography

Author	Study type	Number of patients	Mode	Findings
Chen et al. [38]	Follow-up for five years	24 patients, all children	Resting ECG	ventricular arrhythmias were observed
Mah et al. [39]	Cross-sectional	274 patients, adults and children	2D echo and Ambulatory ECG	7 % had ventricular arrhythmia
Yetman et al. [29]	Follow-up for six years	70 patients, adults and children	2D echo, Resting E.C.G., and Ambulatory ECG	21% had ventricular arrhythmia, 6% had non-sustained ventricular tachycardia

Mitral Valve Prolapse

Mitral valve prolapse is another commonly associated condition seen in patients with MFS. The normal annular height to commissural width ratio (AHCWR) is shown to be around a mean value of 24%. An increase in value indicates more saddle shape and lesser value indicates a planar shape [40]. The normal orientation of the annulus is saddle-shaped as it confers mechanical advantage by adding curvature [41]. A study regarding the three-dimensional structure of the mitral valve and annulus morphology in cases of MFS by Jolley et al. suggested that the AHCWR was reduced in patients with MFS, which was shown to be around a mean value of 20% thus giving it a planar configuration [42]. It has also been shown that the mitral valve leaflets undergo myxomatous thickening and elongation in response to *FBN1* gene mutation [43]. A randomized control trial by Lacro and colleagues suggests that the prevalence of mitral valve prolapse and regurgitation is more in females compared to males, being 45% vs 33% in case of prolapse and 25% vs 13% in case of regurgitation [44]. However, another study by Detaint et al. suggested an equal prevalence of prolapse and regurgitation in both genders [26]. It was also shown that the incidence of these complications increases as age increases [26]. The mechanism for the prolapse and subsequent regurgitation has also been attributed to increased TGF beta activity similar to that seen in aortic root dilation [45]

Post-Bentall Graft Infection

This is a rare yet serious complication in patients undergoing Bentall graft operation, which has been discussed below. This condition arises due to the prosthetic graft getting infected by gram-positive cocci [46]. The diagnosis is done by a combination of clinical features, radiological findings, and blood reports. It is a late-onset condition in which the patient presents with features like prolonged fatigue and fever. Transthoracic echo is suggested in such cases to assess the anatomy of the ventricles and the atrium, which reveals dilation of the left atrium and ventricle [47]. Blood reports show elevated leukocytosis and raised C reactive protein (CRP) levels [47].

Management

As mentioned above, the most common and fatal cardiac manifestation in a case of MFS is aortic root dilation. Therefore, medical management in these cases includes four main drugs, namely calcium channel blockers, whose action is relaxing the smooth muscles by blocking the calcium channels; angiotensin-converting enzyme inhibitors, which act by inhibiting angiotensin 2 production; angiotensin receptor blockers, which act by inhibiting angiotensin 2 type 1 receptors and thus prevent activation of the renin-angiotensin-aldosterone system (RAAS) to promote diuresis; and beta-blockers, which cause myocardial relaxation by blocking beta-1 receptors. These drugs are believed to delay and prevent aortic root dilation [48]. The most commonly used and accepted drug regimen is a combination of beta-blockers supplemented with angiotensin receptor blockers (ARBs) [49]. In a study in 1970 on a standard model of an aorta constructed with Tygon tubing (Saint-Gobain Corporation, Courbevoie, France) and dog aorta, it was suggested that beta-blockers reduced the pressure impulse (dp/dt), which led to limitation of aortic dissection propagation and so is used as a basis of use in humans where it reduces the impact of hemodynamic forces on the aorta [50]. In the case of the ARB, it was shown that RAAS. system activation leading to the release of angiotensin 2 led to an increase in TGF beta. This caused exacerbation of aortic aneurysm and dissection and thus formed the basis for the use of ARB [49].

Regarding surgical options, the most effective procedure is aortic root replacement. It is of two types, namely total root replacement (TRR) and valve-sparing root replacement (VSRR) [23]. TRR was introduced by Bentall and De Bono and involves the replacement of the whole of the aortic root with a prosthetic graft along with mechanical valves as a replacement for the organic valves of the aorta [23]. In the case of post-surgical graft infection, surgical removal of the infected graft is the preferred choice of treatment [47]. However, in patients not fit for surgery, prolonged antibiotic therapy (>3 months) is advised [47]. VSRR, on the other hand, as the name suggests, is a procedure which spares the native valves in a patient. It is mostly preferred in young patients as TRR puts them on lifelong anticoagulation. This can put them at risk for haemorrhage and thromboembolic complications [23]. TRR, on the other hand, has the advantage that the procedure can be performed irrespective of aorta dimensions or the degree of aortic regurgitation [23].

## Conclusions

MFS, as mentioned above, is an extremely rare disease but with a very characteristic presentation like tall stature with long limbs and similarly long digits. This enables the physicians to make a clinical diagnosis on the basis of clinical signs such as thumbs extending far beyond the edge of their hands when they make a fist. It can cause a plethora of cardiac complications of which the most fatal and commonly encountered is an aortic aneurysm and subsequent dissection. This, however, is not the only problem as a mutation in the fibrils also leads to weakened and less compliant myocardium leading to valvular insufficiencies and resultant heart failure. As a result of heart failure, ventricular arrhythmias can also arise. All these factors lead to mortality and morbidity in the patients. Thus such patients must be identified as soon as possible based on clinical features, and 2010 modified Ghent nosology and intervention, which may be medical or surgical, should be initiated. In the initiation of intervention, whether medical or surgical, factors like age and comorbidities should be kept in mind and treated accordingly.
